# Correction: AlCl_3_@ZnO nanostructured material: an efficient green catalyst for the one-pot solvent-free synthesis of 1,4-dihydropyridines

**DOI:** 10.1039/d3ra90088f

**Published:** 2023-09-13

**Authors:** Santosh T. Shinde, Kaluram G. Kanade, Ramesh B. Gawade, Vikram B. Hinge, Manish D. Shinde, Digambar B. Bankar, Nitin M. Thorat, Dinesh P. Amalnerkar

**Affiliations:** a Post Graduate Department of Chemistry and Research Centre, Annasaheb Awate College Manchar-410503 India drsantoshinde@gmail.com; b Centre for Materials for Electronic Technology (C-MET) Off Pashan Road Panchwati Pune-411008 India; c Post Graduate Department of Chemistry and Research Centre, R. B. Narayanrao Borawake College Shrirampur-413709 India; d Post Graduate Department of Chemistry and Research Centre Maharaja Jivajirao Shinde Mahavidyalaya, Shrigonda Ahmednagar-413701 India; e Department of Technology, Savitribai Phule Pune University Pune-411007 India

## Abstract

Correction for ‘AlCl_3_@ZnO nanostructured material: an efficient green catalyst for the one-pot solvent-free synthesis of 1,4-dihydropyridines’ by Santosh T. Shinde *et al.*, *RSC Adv.*, 2023, **13**, 24767–24776, https://doi.org/10.1039/D3RA04277D.

The authors regret that incorrect details were given for ref. 10 in the original article. The correct version of ref. 10 is given as ref. 1 below.

The Royal Society of Chemistry apologises for these errors and any consequent inconvenience to authors and readers.

## Supplementary Material
